# Association of postural education and postural hygiene with low back pain in schoolchildren: Cross-sectional results from the PEPE study

**DOI:** 10.34172/hpp.2023.19

**Published:** 2023-07-10

**Authors:** Aina Maria Galmes-Panades, Pere Antoni Borràs, Josep Vidal-Conti

**Affiliations:** Physical Activity and Sport Sciences Research Group (GICAFE), Institute for Educational Research and Innovation (IRIE), University of the Balearic Islands, Palma, ES-07122, Spain

**Keywords:** Low back pain, Child, Health education, Posture

## Abstract

**Background:**

Low back pain (LBP) is the leading cause of disability in the world that affects the population of all ages globally. The main aim of the present study was to analyze the cross-sectional association of postural education and postural hygiene habits with LBP, differentiating between frequency and intensity of pain.

**Methods:**

This cross-sectional study assessed 849 students aged 10-12 years from primary schools. The study was based on four different structured and self-administered questionnaires: back pain questionnaire, Back Pain and Body Posture Evaluation Instrument (BackPEI), Frequency of Commuting to and from School Questionnaire, and the Hebacaknow questionnaire. In addition, height and weight were included to determine the body mass index (BMI).

**Results:**

Participants with prevalence of LBP were significantly older (*P*=0.038), girls (*P*<0.001), taller (*P*=0.018), and practice active travelled back from school (*P*=0.016). Otherwise, participants with no prevalence of LBP sat correctly at the desk (*P*<0.001). Higher knowledge of postural education was associated with lower intensity of LBP (β=-0.07, CI=-0.12 – -0.02, *P*=0.004).

**Conclusion:**

The knowledge related to postural education it is associated with less LBP. School interventions improving knowledge about postural education, ergonomics and postural hygiene are needed to address this important matter.

## Introduction

 Low back pain (LBP) is the leading cause of disability in the world, and it is on the rise.^1^ The frequency of non-specific LBP in children under the age of 7 is quite low (1%),^2^ however, from the age of 13, with the onset of adolescence, the values are like those of adults, with a range of lifetime prevalence from 27.3% to 74%.^3^

 The cause of LBP is multifactorial, associated with a host of risk factors. According to Foltran et al school environment can expose children to many of them, such as inadequate furniture, heavy backpacks, or prolonged periods of sedentary postures.^4^ However, in accordance with Yamato et al there are no clear evidence that carrying a heavy backpack affects negatively to LBP.^5^ Poor posture can cause many health problems: neck tension, muscle aches, poor circulation, physical and mental stress lack of sleep and pain. To prevent the above health problems, it is important to adapt school environment to environmental needs, increase back care knowledge and correct postural hygiene.^6^ A solid understanding of those concepts helps prevent school place injuries by adjusting tools (desks, chairs, computer screen, etc) to the user, with an emphasis on suitability posture to reduce the effects of repeated movements and prolonged sitting periods.

 There are many factors that increase the risk of LBP, but the vast majority are related to lifestyle habits and postural education and postural hygiene. In a recent study,^7^ variables associated with a high frequency of LBP were posture lying, daily use of tablets and cell phones and computer use. The study by de Souza Santos et al,^3^ identifies that backpack weight and screentime increase the intensity and frequency of LBP in children aged 6-12 years old.

 According to the review of the existing literature,^8,9^ studies are needed to analyze the risk factors for LBP in children, especially in the school environment, and to promote the prevention of these risk factors, in a multifactorial manner, through physical exercise to improve physical condition, postural education and postural hygiene, among others.

 Although it is true that some studies have analyzed the association between knowledge of postural habits and postural hygiene with LBP, there are limitations in this regard. Few studies have focused on the school population,^10^ and there are important biases in some of them, for example, some studies have been carried out with small samples that make it difficult to generalize the results and have carried out very short interventions,^11^ which make it difficult to assess the long-term effects of the intervention carried out.

 There are many studies carried out in school children that study the association between the use of backpack and LBP.^12,13^ A recent systematic review and meta-analysis^14^ conclude that wearing a backpack induces postural changes while standing and affects gait in children and adolescents; however, almost all the changes are not related to the backpack weight. Moreover, there is no clear evidence on the effect of backpacks on LBP,^15-17^ so other factors (anatomical, physiological, or environmental) might play an important role in pain perception.^13^ Consequently, more studies are needed to analyze both the association of backpack weight and the way it is carried with LBP.

 Furthermore, there may be a relationship between these two gaps in knowledge, that is, it is possible that the weight of the backpack is a mediator in the association between the type of transport to school and LBP, since although it is a healthy habit to perform an active commuting to school if during this commuting an overweight backpack is carried, it may have negative effects on LPB. For all these reasons, more studies are needed to analyze both associations separately, and the possible mediating role of the backpack’s weight and the way it is carried in this association.

 The main hypothesis of the present study is that high levels of knowledge of postural education, active commuting to school and better postural hygiene will be associated with less LBP, both in terms of pain frequency and intensity. The aim of the present study is to analyze the cross-sectional association between anthropometric measurements, postural education knowledge, type of commuting to school and postural hygiene habits with LBP.

## Materials and Methods

###  Participants

 This cross-sectional study assessed students aged 10-12 years from primary schools (5th- and 6th-grade) from Majorca (Spain). Data collection was carried out between February and March 2021. The final sample was 849 participants, and according to the formula S = ((z^2^ xp (1 – p)) / e2) / (1 + ((z^2^ xp (1 – p) / e^2^ N)), where S = sample size, N = population size, e = margin of error, z = z-value, and a reliability level of 95%, the sampling error was 3.4%. The sample was obtained from 10 primary schools, of whom 400 were boys (47.1%) and 449 were girls (52.9%), with a mean age of 11.3 (33.5% were 10 years old, 47.9% were 11 years old and 18.6% were 12 years old). The sample was selected from different clusters (schools) using convenience sampling. All schools received a letter inviting them to participate in the study and informing them about the characteristics and objectives of the study.

###  Selection criteria

**Figure 1:**
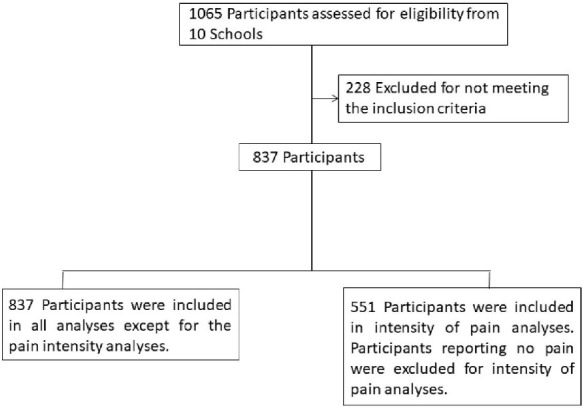
Flow of participants in the PEPE study

 The inclusion criteria were as follows: students must be aged between 10 and 12 years old and attending 5th or 6th grade primary school. Exclusion criteria were as follows: students whose parents or tutors did not return the informed consent form signed and those who did not participate due to illness or disability (see Figure 1).

###  Instruments

 The study was based on four different structured and self-administered questionnaires. The language used in the questionnaires was Spanish. In addition, height and weight were included to determine the body mass index (BMI).

 The data related to back pain was obtained using a validated questionnaire^18^ whose kappa values ranged from 0.88 to 1, and the intraclass correlation coefficient (ICC) values from 0.83 to 0.88. The questionnaire included lifetime prevalence, last 7-days prevalence, point prevalence, LBP in bed or upon rising, LBP impeding usual activities, last 3-months LBP intensity (visual analogue scale ranged from 0 to 10), and also included sex and age.

 The lifetime prevalence of LBP was collected through a self-reported questionnaire, with the question “Have you ever had low back pain?” Initially the response options were five: (1) never, (2) only once, (3) several times, (4) frequently, (5) almost always. To analyze the data, the lifetime prevalence of LBP variable was changed to dichotomous, unifying participants who had never experienced LBP and those who had only experienced pain once in the “No” category and unifying participants who had experienced pain several times, frequently or almost always in the “Yes” category.

 Postural hygiene was assessed using the Spanish version^19^ of the Back Pain and Body Posture Evaluation Instrument (BackPEI),^20^ a self-report questionnaire designed to evaluate back pain and its associated risk factors in school-age children. In the validation study, the kappa coefficient for questions 1–20 in the questionnaire, 5 were classified as “very good”, 8 as “good”, 1 as “moderate”, and 1 as “fair”, and the ICC was 0.951.

 The data included sitting position when writing, sitting position on a chair when talking, sitting position when using a computer, and position adopted when lifting an object from the floor. BackPEI was scored according to the general scoring system to BackPEI.^21^ Each item was coded as 0 = incorrect and 1 = correct. A sum score was computed from the 6 items, namely, the daily postural habits score (range from 0 to 6), so that the higher the score the healthier daily habits related to LBP.

 Variables related to commuting to school, as mode of commuting from and to school, and self-report time from home to school were assessed using the Frequency of Commuting to and from School Questionnaire,^22^ a self-reported questionnaire designed to assess this behavior in Spanish youths. there were two questions: (1) the usual mode of commuting to school, (2) the usual mode of commuting from school. Each question provided these answers: walk, cycle, car, motorcycle, bus or other (in this case, the mode was required), and subsequently were grouped into active commuting, motorized commuting and mixed commuting. The time from home to school and from school to home were reported for each participant in the questionnaire. The response options were less than 10 minutes, between 10 and 20 minutes, more than 20 minutes.

 The Hebacaknow questionnaire^23^ was used to assess the knowledge level of postural education. In the validation study, the Cronbach’s alpha for the 24 items was 0.82, and the ICC was 0.76 for the total score. The resulting 24 multiple choice items were associated with one of the following categories according to conceptual knowledge: topographical-anatomical knowledge; functional–anatomical knowledge; habits in standing posture; or seated; or lying; habits in carrying heavy objects in a backpack; and how to move heavy loads. The score for each item was 0 (wrong option) or 1 (correct option). The scores for each category and for the total questionnaire were obtained computing the mean value of the items involved.

###  Procedure

 The questionnaires were administered at school or home. Teachers gave away the questionnaires at the school’s classroom using laptops or provided families with the guide to fill it. The questionnaires were available on Google Forms. All participants (students, teachers, and parents) were informed about the purpose of the study and its procedure. Moreover, students’ parents or tutors were requested to give their consent for children to participate in the study.

###  Statistical analysis

**Figure 2:**
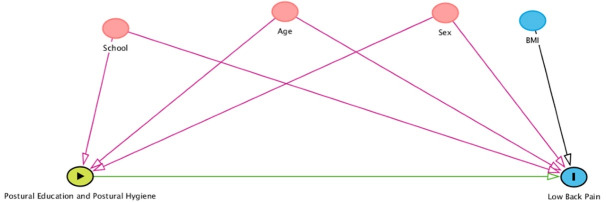
Directed acyclic graphs. Figure legend: The total unconfounded effect of postural education and postural hygiene on Low back Pain, drawn and analysed using DAGitty (http://www.dagitty.net/). The minimal sufficient adjustment sets for estimating the total effect of postural education and postural hygiene on low back pain was age, sex and school. Abbreviations: BMI, body mass index

 Participants were classified in two categories, depending of the presence or absence of LBP. First category included participants with prevalence of LBP (Yes), and the second category those participants without LBP (No). Descriptive characteristics were summarized as means and standard deviations (SDs) or as numbers and percentages (%). One-way analysis of variance (ANOVA) and chi-square test (χ^2^) were used to assess differences across groups of presence of LBP for continuous and categorical variables respectively. Multivariate linear regression analyses were used to estimate the β-coefficients and 95% confidence intervals (CIs) for the associations between intensity of LBP (outcome variable) and weight, height, BMI, knowledge of postural education, types and duration of commuting to school and postural hygiene (exposure variables). Our models were adjusted by the minimally sufficient adjustment set for estimating the total effect of postural education and postural hygiene on LBP, determined using directed acyclic graphs implemented in DAGitty software^24^ available free on http://www.dagitty.net. Therefore, our main models were adjusted for age, sex and school (see Figure 2). Logistic regression models were used to assess the association between categories of pain frequency (ever, in the las 7 days, today and in bed), consequences of pain (daily restrictions) and knowledge of postural education with types and duration of commuting to school and postural hygiene. Receiver operating characteristic (ROC) curve analysis under covariables was used to assess the accuracy of postural hygiene and types of commuting to school for prediction of LBP intensity, consequences of pain, and knowledge of postural education.

 Sensitivity analyses were also conducted, adjusting all statistical analysis for: age, sex, school and BMI. Statistical analyses were performed using Stata v13.0 program. *P* values < 0.05 were deemed as statistically significant.

## Results

Table 1 presents a comparison of participants’ characteristics among the two categories of prevalence of LBP. Participants with prevalence of LBP were significantly older, girls, taller, and practice active travelled to school. Otherwise, participants with no prevalence of LBP sat correctly at the desk (*P*< 0.001). Table S1 (Supplementary file 1) presents a comparison of participants’ characteristics among the two categories of prevalence of LBP in the last 7 days. In line with results of Table 1, participants with prevalence of LBP in the last 7 days were girls and sat incorrectly at the desk (*P*< 0.05).

**Table 1:** Baseline characteristics of the study population across categories of frequency of Life-time prevalence of low back pain

	**Life-time prevalence of low back pain**			
	**Total n**	**Yes**	**No**	***P*** ** value**
Age, years	797	11.3 (0.69)	11.2 (0.67)	**0.038**
Gender	837			**<0.001**
Women, n (%)	443 (52.9)	231 (59.8)	212 (47.0)	
Men, n (%)	394 (47.1)	155 (40.2)	239 (53.0)	
Anthropometric measures				
Weight (kg)	770	43.0 (10.4)	41.6 (9.24)	0.051
Height (cm)	749	151 (9.19)	149 (8.70)	**0.018**
BMI (kg/m^2^)	708	19.9 (3.61)	18.7 (3.56)	0.392
Knowledge of postural education				
Knowledge, score (0-24)	837	9.68 (3.86)	9.65 (3.85)	0.908
Types of commuting to school				
To go to school	837			0.399
Active commuting, n (%)	389 (46.5)	187 (48.5)	202 (44.8)	
Motorised commuting, n (%)	415 (49.6)	182 (47.2)	233 (51.7)	
Mixed commuting, n (%)	33 (3.94)	17 (4.40)	16 (3.55)	
To come back from school	837			**0.016**
Active commuting, n (%)	401 (47.9)	195 (50.5)	206 (45.7)	
Motorised commuting, n (%)	400 (47.8)	168 (43.5)	232 (51.4)	
Mixed commuting, n (%)	36 (4.3)	23 (5.96)	13 (2.88)	
Duration of commuting	837			0.746
Less than 10 minutes, n (%)	546 (65.2)	252 (65.3)	294 (65.2)	
Between 10 and 20 minutes, n (%)	256 (30.6)	120 (31.1)	136 (30.2)	
More than 20 minutes, n (%)	35 (4.18)	14 (3.63)	21 (4.66)	
Postural hygiene				
Sitting at a desk, correct, n (%)	173 (20.7)	56 (14.5)	117 (25.9)	**<0.001**
Sitting in a chair, correct, n (%)	142 (17.0)	64 (16.6)	78 (17.3)	0.784
Sitting in front of a computer, correct, n (%)	328 (39.2)	147 (38.1)	181 (40.1)	0.545
Picking up an object, correct, n (%)	208 (24.9)	102 (26.4)	106 (23.5)	0.330

Abbreviations: BMI; body mass index. Data shown is mean (standard deviation), unless otherwise specified.

Table 2 shows the β-coefficients (95% CIs) for the associations between intensity of LBP and anthropometric measures and knowledge of postural education. Higher knowledge of postural education was associated with lower intensity of LBP (β = -0.07, CI = -0.12 - -0.02, *P* = 0.004). No significant associations were found with weight, height and BMI.

**Table 2:** Associations of anthropometric measures and postural education knowledge with intensity of low back pain

**Exposures**	**Outcome (intensity of low back pain, continuous)**		***P*** ** value**
Anthropometric measures			
Weight (kg)	Model 1	-0.004; (-0.023; 0.015)	0.651
	Model 2	-0.034; (-0.073; 0.004)	0.085
Height (cm)	Model 1	-0.019; (-0.041; 0.004)	0.099
	Model 2	-0.018; (-0.040; 0.005)	0.121
BMI (kg/m^2^)	Model 1	0.023; (-0.031; 0.077)	0.396
	Model 2		
Knowledge of postural education			
Knowledge, score (1-24)	Model 1	-0.070; (-0.117; -0.022)	**0.004**
	Model 2	-0.039; (-0.090; 0.011)	0.125

Values shown are β (95% CI). Abbreviations: BMI; Model 1, adjusted for: age, sex and school; model 2, adjusted for: age, sex, school and BMI. Sample included in the analysis, n = 551. The values 0 have been eliminated, with the aim of analysing what may be associated with greater back pain, in cases in which back pain already exists.

Table 3 shows the β-coefficients (95% CIs) for the associations between intensity of LBP in continuous and types and duration of commuting to school and postural hygiene by categories. No statistically significant associations were found.

**Table 3:** Associations of types of commuting to schools and postural hygiene with intensity of low back pain

**Exposures**	**Outcome** **(Intensity of low back pain, continuous)**	***P *** **for trend**
**Types of commuting to school**		
To go to school		0.391
Active commuting	0 (Ref.)	
Motorised commuting	-0.08 (-0.46;0.29)	
Mixed commuting	-0.40 (-0.46;0.29)	
To come back from school		0.782
Active commuting	0 (Ref.)	
Motorised commuting	-0.16 (-0.53;0.21)	
Mixed commuting	0.20 (-0.59;0.99)	
Duration of commuting		0.738
Less than 10 minutes	0 (Ref.)	
Between 10 and 20 minutes	0.02 (-0.35;0.40)	
More than 20 minutes	0.20 (-0.70;1.10)	
**Postural hygiene**		
Sitting at a desk, incorrect	0 (Ref.)	
Sitting at a desk, correct	0.11 (-0.34;0.56)	0.627
Sitting in a chair, incorrect	0 (Ref.)	
Sitting in a chair, correct	-0.06 (-0.51;0.39)	0.799
Sitting in front of a computer, incorrect	0 (Ref.)	
Sitting in front of a computer, correct	-0.01 (-0.36;0.35)	0.967
Picking up an object, incorrect	0 (Ref.)	
Picking up an object, correct	-0.09 (-0.48;0.31)	0.661

Values shown are β (95% CI). Model 1, adjusted for: age, sex, and school. Sample included in the analysis, n = 551. The values 0 have been eliminated, with the aim of analysing what may be associated with greater back pain, in cases in which back pain already exists.

Table 4 shows the odds ratios for types and duration of commuting to school and postural hygiene by categories of pain frequency, consequences of pain and knowledge of postural education. Sitting correctly at a desk was significantly associated with no LBP ever (OR = 1.98, CI = 1.37-2.86, *P* < 0.001), nor in the last 7 days (OR = 1.70, CI = 1.06-2.73, *P* = 0.028). In addition, to come back from school actively and picking up correctly an object were associated with increased knowledge of postural education (OR = 0.52, CI = 0.34-0.81, *P* = 0.004 and OR = 0.58, CI = 0.35-0.94, *P* = 0.027, respectively).

**Table 4:** Odds Ratio of postural education and hygiene according to low back pain

**Exposures**	**Outcome**											
	**Pain frequency**								**Consequences of pain**		**knowledge of postural education**	
	**LBP ever (no)**	***P*** ** for trend**	**LBP in the last 7 days (no)**	***P*** ** for trend**	**LBP today (no)**	***P*** ** for trend**	**LBP in bed (no)**	***P*** ** for trend**	**LBP daily restrictions**	***P*** ** for trend**	**Knowledge, score (0-24)**	***P*** ** for trend**
Commuting to school			
To go to school active	0.94 (0.69; 1.28)	0.683	1.07 (0.74; 1.55)	0.716	1.04 (0.63; 1.73)	0.880	1.22 (0.78; 1.90)	0.388	0.99 (0.72; 1.37)	0.971	0.68 (0.44; 1.06)	0.091
To come back from school actively	0.75 (0.55; 1.02)	0.066	0.89 (0.61; 1.28)	0.519	1.12 (0.68; 1.86)	0.654	1.02 (0.66; 1.58)	0.923	0.96 (0.70; 1.32)	0.821	0.52 (0.34; 0.81)	**0.004**
Duration of commuting	0.92 (0.68; 1.25)	0.580	0.82 (0.57; 1.19)	0.297	0.96 (0.57; 1.60)	0.873	1.09 (0.70; 1.69)	0.709	1.21 (0.88; 1.67)	0.247	1.28 (0.85; 1.94)	0.240
Postural hygiene				
Sitting correctly at a desk	1.98 (1.37; 2.86)	**<0.001**	1.70 (1.06; 2.73)	**0.028**	0.93 (0.50; 1.71)	0.811	1.25 (0.72; 2.16)	0.432	1.16 (0.80; 1.70)	0.427	1.19 (0.74; 1.91)	0.480
Sitting correctly in a chair	0.99 (0.68; 1.45)	0.977	1.46 (0.89; 2.38)	0.131	1.53 (0.76; 3.10)	0.233	1.33 (0.74; 2.40)	0.338	0.99 (0.66; 1.46)	0.941	1.42 (0.87; 2.34)	0.165
Sitting correctly in front of a computer	1.02 (0.76; 1.37)	0.902	1.24 (0.87; 1.78)	0.240	1.26 (0.76; 2.10)	0.363	1.17 (0.77; 1.80)	0.461	0.95 (0.70; 1.29)	0.737	1.19 (0.80; 1.77)	0.390
Picking up correctly an object	0.85 (0.61; 1.18)	0.334	0.87 (0.59; 1.29)	0.491	1.00 (0.57; 1.75)	0.995	0.87 (0.54; 1.38)	0.545	0.99 (0.70; 1.40)	0.949	0.58 (0.35; 0.94)	**0.027**

Odds ratios values are shown (95% CI). Logistic regression models were adjusted for age, sex, and school. Abbreviations: LBP; low back pain. Variables categorization – Exposures: Go to school and come back to school, the values shown correspond to active commuting, reference category was inactive commuting. Duration of commuting, the values shown correspond to more than 10 minutes, reference category was less than 10 minutes. Postural hygiene variables, the values shown correspond to correct postures, reference category was incorrect postures. Variables categorization – Outcomes: The values shown correspond to no pain and no impediments respectively, reference categories were presence of pain and impediments respectively. The knowledge variable was presented in two categories, the first for values between 0-9 (reference category) and the second for values between 10-24 (values shown).

Table 5 shows the AUC - area under ROC curve, for different postural education and hygiene for elevated LBP risk. Sitting correctly at a desk was significantly associated with no LBP (AUC = 0.56, CI = 0.52-0.60, *P* = 0.004). Active commuting to school was border associated with higher knowledge of postural education (*P* = 0.051).

**Table 5:** Adjusted area under the ROC curve analysis for postural education and hygiene according to low back pain

**Exposures**	**Outcome**											
	**Pain Frequency**								**Consequences of pain **		**knowledge of postural education**	
	**LBP ever (no)**	***P *** **value**	**LBP in the last 7 days (no)**	***P *** **value**	**LBP today (no)**	***P *** **value**	**LBP in bed (no)**	***P *** **value**	**LBP daily restrictions**	***P *** **value**	**Knowledge, score (0-24)**	***P *** **value**
Commuting to school												
To go to school active	0.52 (0.48;0.56)	0.446	0.50 (0.45; 0.55)	0.931	0.50 (0.43; 0.57)	0.967	0.52 (0.46; 0.57)	0.553	0.49 (0.45; 0.53)	0.621	0.54 (0.50; 0.58)	0.051
To come back from school actively	0.52 (0.48; 0.56)	0.449	0.50 (0.45; 0.55)	0.979	0.50 (0.43; 0.57)	0.943	0.53 (0.47; 0.58)	0.391	0.48 (0.44; 0.52)	0.380	0.53 (0.50; 0.57)	0.086
Duration of commuting	0.49 (0.45; 0.53)	0.715	0.51 (0.46; 0.56)	0.725	0.52 (0.45; 0.59)	0.558	0.52 (0.47; 0.58)	0.435	0.52 (0.48; 0.56)	0.392	0.49 (0.45; 0.53)	0.711
Postural hygiene												
Sitting correctly at a desk	0.56 (0.52; 0.60)	**0.004**	0.46 (0.41; 0.51)	0.095	0.49 (0.42; 0.56)	0.755	0.47 (0.42; 0.53)	0.354	0.52 (0.48; 0.56)	0.356	0.49 (0.45; 0.53)	0.721
Sitting correctly in a chair	0.51 (0.47; 0.54)	0.808	0.47 (0.43; 0.52)	0.261	0.48 (0.42; 0.55)	0.614	0.48 (0.42; 0.53)	0.402	0.50 (0.46; 0.54)	0.881	0.51 (0.47; 0.54)	0.807
Sitting correctly in front of a computer	0.51 (0.47;0.55)	0.609	0.47 (0.42; 0.52)	0.242	0.47 (0.40; 0.54)	0.376	0.49 (0.44; 0.55)	0.831	0.50 (0.46; 0.54)	0.943	0.48 (0.44; 0.52)	0.376
Picking up correctly an object	0.49 (0.45; 0.53)	0.466	0.52 (0.47; 0.53)	0.531	0.49 (0.43; 0.56)	0.858	0.51 (0.46; 0.57)	0.645	0.50 (0.46; 0.54)	0.956	0.47 (0.43; 0.50)	0.080

AUC, area under receiver operating characteristic (ROC) curve. TThe AUCs and CIs of different postural education and higyene for elevated LBP risk are shown. Abbreviations: LBP; low back pain. Variables categorization – Exposures: Go to school and come back to school, the values shown correspond to active commuting, reference category was inactive commuting. Duration of commuting, the values shown correspond to more than 10 minutes, reference category was less than 10 minutes. Postural hygiene variables, the values shown correspond to correct postures, reference category was incorrect postures. Variables categorization – Outcomes: The values shown correspond to no pain and no impediments respectively, reference categories were presence of pain and impediments respectively. The knowledge variable was presented in two categories, the first for values between 0-9 (reference category) and the second for values between 10-24 (values shown).

 The results were relatively consistent when linear and logistic regression analyses were further-adjusted for BMI.

## Discussion

 Although there are many studies that link LBP with knowledge related to health and specifically with back health, there is very little literature related to the knowledge and health habits of children and adolescents and their relationship with LBP.^25^ The present study analyses the association between BMI, postural hygiene, type of commuting to and from school (motorized vs active), and the degree of knowledge of postural education, with LBP prevalence, intensity and frequency in children aged 10 to 12 years. The aim is to provide information that will help future intervention protocols.

 Results from the present study are consistent with previous research, where LBP being more prevalent in girls, taller schoolchildren, older schoolchildren, and those who sit poorly at the desk.^26^ However, according to the present results, schoolchildren who travelled using motorized transport had lower levels of LBP.^27^ Although not analyzed in the present study, the greater presence of LBP in those schoolchildren who walk to school could be related to carrying backpacks for longer periods of time, bearing in mind that it is common for backpacks to exceed the recommended weight, and not to be carried in the correct or ergonomic position for the back,^28^ although there is controversy in the results regarding the impact of schoolbags into LBP in children and adolescents and some authors consider negative evidence of backpack or schoolbag weight and LBP in children and adolescents.^29^ Active commuting to school, has been associated with higher levels of total daily physical activity, but not to higher levels of physical fitness.^30^ On the contrary, physical fitness is associated with better levels LBP.^8^

 Promoting a higher level of physical fitness has to be on the scope of future interventions to prevent and improve LBP. Furthermore, active commuting to school is a tool for reducing sedentary time and introducing physical activity between sedentary periods, which seems to have beneficial effects on the overall health of schoolchildren.^31^ Although there is a growing body of literature investigating active commuting to school, to the best of our knowledge, there are no studies examining the association between type of commuting to school and LBP.^32,33^ A separate chapter deserves the necessary work to be done in improving knowledge of the use of the school backpack. The present study is part of the PEPE study, where the possible effect of backpack weight on LBP will be analyzed. Unfortunately, this data was not yet available for inclusion in the present analyses.

 The present results indicate that the prevalence of LBP is lower in children who present better results in postural habits and knowledge of postural hygiene, the main example is the posture of sitting at the desk, in concordance with the findings of de Souza Santos et al.^3^ It may be that the only significant effect in terms of sitting position has been observed in “sitting correctly at the desk” due to the fact that it is the most repeated position during school hours, and therefore, the one that could be most emphasized at school, i.e. the one on which teachers have given the most feedback. This element indicates the way with respect to possible school interventions in improving knowledge about postural education, ergonomics, and postural hygiene, on the contrary, factors such as gender, height or age are not modifiable. On the other hand, by knowing that these are risk factors for developing LBP, specific interventions can be made for this population groups. That is, taller children, girls, and to emphasize early in life so that habits are well acquired in pre-adolescence and adolescence, when pain is most likely to manifest itself.

 As regards knowledge of postural education, it is particularly important that it is associated with less LBP. This may suggest that in order to prevent LBP, the population should be encouraged to have adequate knowledge of postural education from childhood and adolescence. Postural education could be considered part of the Health literacy, defined by the WHO as ‘the cognitive and social skills which determine the motivation and ability of individuals to gain access to understand and use information in ways which promote and maintain good health.^34^ In order to obtain better levels of health literacy in our school children, studies show that a greater effort is required to make health education programs easier to understand^35^; carrying out targeted interventions to improve health literacy, and also target those interventions to specific objectives such as, knowledge of Postural Education and hygiene. The findings of this study means that future interventions may focus on health literacy related to LBP knowledge and be primary addressed to girls.

 A marked strength of this study was the use of a large sample of girls and boys in school age, from 10 different schools, combining schools from different geographical areas, which increases the representativeness of the sample. All questionnaires were validated and extensively used in this population. Moreover, sophisticated statistical analyses where performed. Furthermore, the exposure variables represent an innovative aspect in LBP research and may establish the basis for future interventions in the child and adolescent population, favoring health education, the promotion of lifelong healthy habits, and prevention as an educational and therapeutic tool. All this will help society to be more responsible with its health and self-care and may have important implications for health systems in terms of savings and cost-effectiveness.

 In terms of limitations, the cross-sectional design of the study does not allow causality to be established. Furthermore, the postural hygiene variable is self-reported, which may lead to bias. The scarcity of significant results limits the ability to draw firm conclusions. A certain level of recall bias can be assumed caused by differences in the accuracy or completeness of memories retrieved by study participants regarding past events and experiences. There is some loss of data due to the ages of the subjects who in the school environment usually have a high level of non-appearance due to illness.

## Conclusion

 Results from this cross-sectional study indicate that LBP is more prevalent in girls, taller schoolchildren, older schoolchildren, those who sit poorly at the desk and those who walk to school. On the contrary, knowledge related to postural education is associated with less LBP in this school population. School interventions in improving knowledge about postural education, ergonomics and postural hygiene are needed to address this important matter. In addition to studies on backpack weight and mode of use, to clarify why students who walk to school have higher LBP than those who use motorized transport.

## Competing Interests

 The authors declare that they have no competing interests.

## Ethical Approval

 The study was approved by the Research Ethics Committee of the University of the Balearic Islands (reference number: 130CER19).

## Funding

 Grant [RTI2018-101023-A-I00] funded by Ministry of Science and Innovation MCIN/AEI/10.13039/501100011033 and by “European Regional Development Fund (ERDF) A way of making Europe”.

## Supplementary Files

Supplementary file 1 contains Table S1.
