# Supplementary material for: Association of postural education and postural hygiene with low back pain in schoolchildren: Cross-sectional results from the PEPE study
Source: Health Promot Perspect. 2023 Jul 10;13(2):157–65. doi: 10.34172/hpp.2023.19 (PMC10439453; doi:10.34172/hpp.2023.19)
Supplement: Supplementary file 1 — contains Table S1. [file hpp-13-157-s001.pdf]

Galmes-Panades et al, **Health Promotion Perspectives,** 2023, 13(2), 157-165
doi: 10.34172/hpp.2023.19
https://hpp.tbzmed.ac.ir

Supplementary file 1

Table S1. Baseline characteristics of the study population across categories of presence of low back pain in the last 7 days

	Presence of low back pain in the last 7 days			
	Total n	Yes	No	P - value
Age, years	797	11.3 (0.72)	11.3 (0.67)	0.754
Women, n (%)	443 (52.9)	106 (60.2)	337 (51.0)	**0.029**
**Anthropometric measures**				
Weight (kg)	770	42.4 (10.2)	42.2 (9.72)	0.871
Height (cm)	749	150 (8.62)	150 (9.04)	0.892
BMI (kg/m^2)	708	19.0 (3.80)	18.7 (3.53)	0.533
**knowledge of postural education**				
Knowledge, score (0-24)	837	9.41 (3.78)	9.73 (3.87)	0.332
**Types of commuting to school**				
To go to school	837			0.860
Active commuting, n (%)	389 (46.5)	83 (47.2)	306 (46.3)	
Motorized commuting, n (%)	415 (49.6)	85 (48.3)	330 (49.9)	
Mixed commuting, n (%)	33 (3.90)	8 (4.50)	25 (3.80)	
To come back from school	837			0.301
Active commuting, n (%)	401 (47.9)	86 (48.9)	315 (47.7)	
Motorized commuting, n (%)	400 (47.8)	79 (44.9)	321 (48.6)	
Mixed commuting, n (%)	36 (4.30)	11 (6.20)	25 (3.70)	
Duration of commuting	837			0.792
Less than 10 minutes, n (%)	546 (65.2)	111 (63.1)	435 (65.8)	
Between 10 and 20 minutes, n (%)	256 (30.6)	57 (32.4)	199 (30.1)	
More than 20 minutes, n (%)	35 (4.20)	8 (4.50)	27 (4.10)	
**Postural hygiene**				
Sitting at a desk, correct, n (%)	173 (20.7)	25 (14.2)	148 (22.4)	**0.017**
Sitting in a chair, correct, n (%)	142 (17.0)	23 (13.1)	119 (18)	0.121
Sitting in front of a computer, correct, n (%)	328 (39.2)	61 (34.7)	267 (40.4)	0.166
Picking up an object, correct, n (%)	208 (24.9)	48 (27.3)	160 (24.2)	0.403

Data shown is mean (SD), unless otherwise specified. Abbreviations: BMI; body mass index.
